# Economic evaluation of return-to-work interventions for mental disorder-related sickness absence: two years follow-up of a randomized clinical trial

**DOI:** 10.5271/sjweh.4012

**Published:** 2022-04-29

**Authors:** Anna Finnes, Jeffrey S Hoch, Pia Enebrink, JoAnne Dahl, Ata Ghaderi, Anna Nager, Inna Feldman

**Affiliations:** 1 Department of Clinical Neuroscience, Karolinska Institutet, Stockholm, Sweden; 2 Department of Health Sciences, University of California, Davis, USA; 3 Department of Psychology, Uppsala University, Uppsala, Sweden; 4 Department of Neurobiology, Care Sciences and Society, Division of Family Medicine and Primary Care, Karolinska Institutet, Stockholm, Sweden; 5 Department of Public Health and Caring Sciences, Uppsala University, Uppsala, Sweden

**Keywords:** acceptance and commitment therapy, ACT, anxiety, common mental disorder, depression, effectiveness, incremental net benefit, RTW, stress, sickness benefit, work disability, workplace intervention

## Abstract

**Objective:**

The objective was to (i) assess the long-term cost-effectiveness of acceptance and commitment therapy (ACT), a workplace dialog intervention (WDI), and ACT+WDI compared to treatment as usual (TAU) for common mental disorders and (ii) investigate any differences in cost-effectiveness between diagnostic groups.

**Methods:**

An economic evaluation from the healthcare and limited welfare perspectives was conducted alongside a randomized clinical trial with a two-year follow-up period. Persons with common mental disorders receiving sickness benefits were invited to the trial. We used registry data for cost analysis alongside participant data collected during the trial and the reduction in sickness absence days as treatment effect. A total of 264 participants with a diagnosis of depression, anxiety, or stress-induced exhaustion disorder participated in a two-year follow-up of a four-arm trial: ACT (N=74), WDI (N=60), ACT+WDI (N=70), and TAU (N=60).

**Results:**

For all patients in general, there were no statistically significant differences between interventions in terms of costs or effect. The subgroup analyses suggested that from a healthcare perspective, ACT was a cost-effective option for depression or anxiety disorders and ACT+WDI for stress-induced exhaustion disorder. With a two-year time horizon, the probability of WDI to be cost-saving in terms of sickness benefits costs was 80% compared with TAU.

**Conclusions:**

ACT had a high probability of cost-effectiveness from a healthcare perspective for employees on sick leave due to depression or anxiety disorders. For participants with stress-induced exhaustion disorder, adding WDI to ACT seems to reduce healthcare costs, while WDI as a stand-alone intervention seems to reduce welfare costs.

Mental health disorders are among the main contributors to the global years lived with disability (1) and are associated with work disability and impaired emotional and social functioning (2, 3) as well as long-term sickness absence (SA) in economically developed countries (3–5). Mental disorders cause direct costs within the healthcare system, but the primary cost driver is indirect costs related to loss of productivity and insurance costs (6). SA due to mental disorders is dominated by depression, anxiety and stress-related disorders (4, 7, 8), which are highly and increasingly prevalent in the working-age population (9).

Evidence-based treatments for depression and anxiety include antidepressant medication and psychotherapy, predominantly cognitive behavior therapy (10); for more effective return-to-work (RTW) after SA, combining workplace changes with a clinical program is suggested (11). For chronic stress-related disorders, treatment effects have so far been marginal (12), which might be due to a general lack of clarity regarding the measurement and definition of stress-related ill-health. Recently, the World Health Organization (WHO) recognized burnout as an official medical diagnosis in the ICD 11^th^ edition, defined as a “syndrome conceptualized as resulting from chronic workplace stress that has not been successfully managed” (13). It is distinct from depression and anxiety diagnoses as it refers specifically to phenomena in the occupational context, which implies that effective treatment strategies may differ from those used to treat depression and anxiety disorders.

Mental health disorders are associated with long periods of sick leave, and the RTW process may be extended over time. Economic evaluations, including cost-effectiveness analyses, can provide important information – including costs and consequences of treatment programs – to aid decision-making in different societal sectors. The value of an economic evaluation has been questioned in cases where RTW interventions fail to show effectiveness (14); however, best practice involves studying cost-effectiveness even when a new intervention is not significantly more effective following the principle that if two treatments are equally effective, then the lowest cost treatment is the treatment of choice (15). Further, longer-term follow-ups are necessary since the first period of absence often is followed by a recurrence resulting in increasing societal cost in the long term (16). So far, most cost-effectiveness analyses of mental health RTW interventions have been based on follow-up periods of up to one year (14, 17, 18).

The objective of the present study was to evaluate the cost-effectiveness of acceptance and commitment therapy (ACT), workplace dialog intervention (WDI), and a combination of ACT and WDI (ACT+WDI) compared to a control group receiving treatment as usual (TAU), for individuals on SA due to common mental disorders. The aims were to (i) estimate the economic attractiveness of the interventions using one- and two-year follow-up data and (ii) explore heterogeneity in the cost-effectiveness between the interventions when randomly provided to patients with either depression or anxiety disorders or with chronic stress-induced exhaustion disorder. The study was undertaken in the region of Stockholm in Sweden.

## Methods

### Study design and participants

The original study was designed as an RCT with 352 participants randomized into four conditions; ACT, WDI, ACT+WDI, and TAU. In the last follow-up assessment, 24 months after randomization, 264 participants agreed to participate. The study was registered at clinicaltrials.gov (Dnr NCT01805583) and approved by the Regional Ethical Review Board in Stockholm (Dnr 2012/2109-31/5). Participants had to fulfill diagnostic criteria for depression (F33), anxiety disorder (F32), or stress-induced exhaustion disorder (F43.8) according to the Swedish version of the International Statistical Classification of Diseases and Related Health Problems (ICD-10). Further, inclusion criteria were: age 18–60 years, employed, and on SA at the time for inclusion in the study. Exclusion criteria were: SA for >12 months, active suicide ideation, severe depression, history of bipolar disorder or psychosis, substance abuse or dependence, unemployment or self-employment, and insufficient comprehension of the Swedish language. More detailed information can be found in earlier publications (19, 20).

### Interventions

Treatments were administered over three months. ACT treatment consisted of six 1-hour sessions with a psychologist, targeting all core ACT therapeutic processes, including acceptance, mindfulness, self as context, defusion, values clarification and, committed action. Homework exercises with relevant content were suggested after every session. WDI-treatment consisted of three steps: (i) the participant interview including open questions regarding the perception of causes of SA and factors that may facilitate RTW, (ii) the supervisor interview regarding causes and facilitators and, (iii) the three-party convergence dialog meeting, aiming at agreeing on a rehabilitation plan. ACT+WDI-treatment combined the two above-mentioned strategies administered by two separate therapists. TAU was defined as regular visits to a general practitioner for administration of SA and any other treatment that is recommended for common mental disorders within the primary care in Sweden.

### Economic evaluation

Data for the cost-effectiveness analysis (21) were collected at pre- and post-treatment, 6, 9, 12, and 24 months follow-up. The analyses time horizons were 12 and 24 months, reflecting the time from randomization to one- and two-years follow-up. Cost-effectiveness analyses were conducted from two perspectives: (i) a healthcare perspective, including intervention costs and healthcare cost of the participants during follow-up periods, and (ii) a limited welfare perspective including the intervention costs and cost for sickness benefits during follow-up periods. All costs were collected in 2015 Swedish krona and converted into 2020 euro (€) using a conversion rate based on purchasing power parities for the gross domestic product (22). The results were presented as incremental cost-effectiveness ratios (ICER), where the difference in costs ΔC was divided by the difference in effects ΔE, comparing the three treatment alternatives to TAU. Additionally, incremental net benefit (INB) (23) estimates were computed using a variety of willingness-to-pay (WTP) values for one avoided SA day.

### Costs

Intervention costs for the treatment alternatives were established in a previously published study (20). Total treatment costs amounted to €297 per participant for ACT, €358 per participant for WDI, and €652 for ACT+WDI. Healthcare resource use was collected at all follow-up assessments using questionnaires where participants were asked to state the number of visits to different health professionals (medical doctor, psychologist/counselor, physical therapist, and nurse) during the follow-up periods. Unit costs (cost per visit) were derived from the Cost-per-Patient Database (Swedish Association of Local Authorities and Regions) and then multiplied by the number of visits. All the unit costs are presented in supplementary material (www.sjweh.fi/article/4012) table S1. Sickness benefits costs were collected from the Swedish Social Insurance Agency (SSIA), comprising the total amount of insurance benefits paid to each participant during follow-up.

### Health outcome

The health outcome of interest was SA days. SA data were obtained from the registry of the SSIA. SA days were calculated for every participant during the one- and two-year follow-ups by adding up full-time and part-time SA to create full-day equivalents. The interventions aimed to reduce SA days; thus a negative difference in health outcome between the intervention and comparison indicates a relative improvement in health outcome. Consequently, an incremental effectiveness estimate of -1 means that a new alternative is associated with one less SA day compared to TAU. Additionally, reduction in SA days is meaningful for decision-makers, and they can establish the WTP intuitively for one avoided SA day (eg, as the average disability payment/day).

### Analyses

Incremental costs and incremental effects for each intervention compared with TAU were estimated using ordinary least squares (OLS). Incremental costs were calculated separately for healthcare costs at every assessment point and for total sickness benefits costs at one- and two-year follow-ups. Incremental effects (reduction in SA days) were calculated at one- and two-year follow-ups. Regression calculations were adjusted for age, sex, education level, birthplace, previous grade of SA and marital status. More details are presented in the online supplementary material.

Missing data for the frequencies of healthcare use were imputed using predictive mean matching (PMM) based on gender, age, level of education, diagnosis, country of origin, and received sick benefits prior to the start of the interventions. Cost-effectiveness results were summarized on cost-effectiveness planes. Confidence intervals (CI) for the estimates were created using non-parametric bootstrapping. The likelihood of each intervention being more favorable compared to TAU was presented using cost-effectiveness acceptability curves (CEAC) across a range of WTP thresholds (24). CEAC represent the uncertainty concerning the cost-effectiveness of an RTW intervention in the context of decisions involving three different interventions. The WTP = 0 describes the probability that an intervention is cost-saving and may have special meaning to decision-makers when they are unwilling to pay for health gains.

All the analyses were conducted from a healthcare perspective and a limited welfare perspective for the whole sample of study participants and separately for two subgroups: (i) participants with stress-induced exhaustion diagnosis (F43.8) and (ii) participants with depression or an anxiety disorder diagnosis (F32 and F41). The results are presented for the whole sample and the subgroups as mentioned above.

## Results

### Baseline characteristics

**Figure 1:**
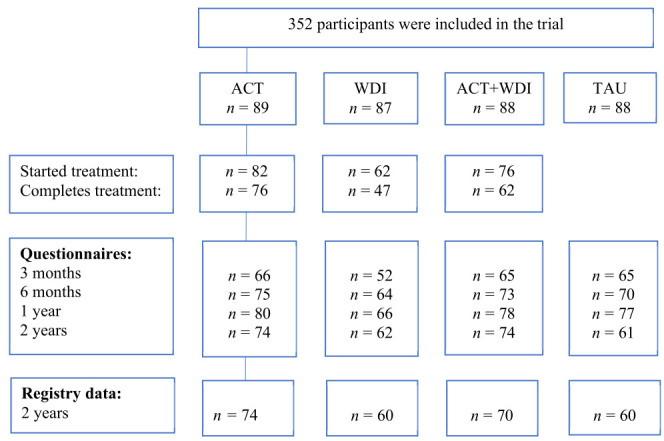
Trial flow diagram.

A trial flow diagram is presented in figure 1. A total of 264 of the original 352 participants consented to data being collected from SSIA registers (ACT=74 [82%], WDI=60 [66%], ACT+WDI=70 [78%], TAU=60 [74%]).

There were no significant differences at baseline between the four groups regarding descriptive data, proportions of disorders, outcome measures, or sickness benefit payment, see table 1. Missing data for frequencies of healthcare use were observed at all follow-ups and are presented in supplementary table S2.

**Table 1:** Characteristics of the patients at pre-measurement. [ACT=acceptance and commitment therapy; NDSA=net days of sickness absence; SD=standard deviation; SED=stress-induced exhaustion disorder; TAU= treatment as usual; WDI=work dialog intervention].

	Total			ACT			WDI			ACT+WDI			TAU			Group diff.
					
	N	%	Mean (SD)	N	%	Mean (SD)	N	%	Mean (SD)	N	%	Mean (SD)	N	%	Mean (SD)	P-value
Age	264		46.8 (8.8)	74		46.2 (8.1)	60		45.4 (9.0)	70		48.6 (8.7)	60		46.7 (9.2)	0.185
NDSA (pre)	264		94.5 (59.7)	74		94.0 (55.2)	60		98.2 (62.5)	70		103 (71.5)	60		81.2 (44.1)	0.117
Benefits in € (12 mths prior)	264		6595 (4223)	74		6467 (3754)	60		6868 (4619)	70		7171 (5070)	60		5808 (3092)	0.225
Women	209	79.2		59	79.7		47	78.3		55	78.6		48	80.0		0.994
University education	162	61.4		48	64.9		33	55.0		43	61.4		38	63.3		0.681
Born in Sweden	212	80.3		61	82.4		47	78.3		54	77.1		50	83.3		0.765
Diagnostic group																
SED	157	59.5		41	55.4		39	65.0		41	58.6		36	60.0		0.729
Depression or anxiety	107	40.5		33	44.6		21	35.0		29	41.4		24	40.0		
Sick listed 75/100%	137	52.1		36	49.3		26	43.3		40	57.1		35	58.3		0.293

### Cost-effectiveness analyses

The results of incremental analyses are presented in table 2.

**Table 2:** Mean differences in costs (in €2020) and outcomes (SA days) between treatment arms. [ACT=acceptance and commitment therapy; CI=confidence interval; SA=sickness absence (negative SA days indicate improvement in health outcome (reduction in sickness absence), more SA days is less favorable); SED=stress-induced exhaustion disorder; TAU=treatment as usual; WDI=work dialog intervention]

		Difference in	ACT vs TAU	WDI vs TAU	ACT+WDI vs TAU
		
			Estimate (95% CI)	Estimate (95% CI)	Estimate (95% CI)
All participants, N=264					
Over 1 year	Cost type	Healthcare	-928.75 (-1586.17‒15.44)	55.25 (-685.86‒1211.44)	-782.01 (-1600.05‒65.92)
		Benefits	209.18 (-1982.08‒2400.45)	862.38 (-1452.58‒3177.35)	1242.89 (-974.98‒3460.77)
		Intervention	297	358	652
	Outcome	SA days	9.84 (-22.48‒42.16)	14.86 (-19.29‒49.00)	24.45 (-8.26‒57.17)
Over 2 years	Cost type	Healthcare	-805.93 (-4480.13‒24.14)	-467.28 (-2616.48‒1599.93)	-863.3 (-3944.10‒102.51)
		Benefits	553.74 (-3644.98‒4752.45)	1815.74 (-2620.00‒6251.49)	1661.95 (-2587.76‒5911.65)
		Intervention	297	358	652
	Outcome	SA days	19.62 (-44.76‒84.01)	34.17 (-33.85‒102.19)	37.29 (-27.88‒102.45)
Participants with depression or anxiety disorder, N=107					
Over 1 year	Cost type	Healthcare	-1726.42 a (-2933.23‒-483.28)	702.36 (-830.64‒2324.99)	-593.87 (-2142.56‒999.14)
		Benefits	1057.32 (-2468.31‒4582.94)	3378.73 (-635.79‒7393.25)	1903.80 (-1733.03‒5540.63)
		Intervention	297	358	652
	Outcome	SA days	21.18 (-32.13‒74.49)	55.68 (-5.02‒116.38)	37.86 (-17.13‒92.85)
Over 2 years	Cost type	Healthcare	-1839.10a (-7984.58‒-110.22)	-1172.08 (-4822.59‒3007.78)	-1920.85 (-7509.29‒777.40)
		Benefits	917.97 (-5947.64‒7783.58)	7636.86 (-180.80‒15454.52)	2945.39 (-4136.78‒10027.55)
		Intervention	297	358	652
	Outcome	SA days	25.08 (-82.83‒132.99)	132.87 ^a^ (9.99‒255.74)	59.72 (-51.59‒171.04)
Participants with stress-induced exhaustion disorder, N= 57					
Over 1 year	Cost type	Healthcare	-380.54 (-1262.71‒575.78)	-375.39 (-1142.65‒494.65)	-1010.13a (-1940.73‒-275.78)
		Benefits	-646.15 (-3527.36‒2235.06)	-1270.81 (-4199.63‒1658.02)	641.06 (-2196.29‒3478.42)
		Intervention	297	358	652
	Outcome	SA days	0.12 (-41.56‒41.79)	-17.95 (-60.31‒24.41)	15.49 (-25.55‒56.53)
Over 2 years	Cost type	Healthcare	9.19 (-1895.32‒1176.56)	107.73 (-1766.70‒980.60)	74.18 (-2494.04‒156.54)
		Benefits	-295.53 (-5660.89‒5069.84)	-2837.14 (-8291.17‒2616.89)	432.61 (-4851.08‒5716.31)
		Intervention	297	358	652
	Outcome	SA days	10.93 (-69.44‒91.29)	-40.89 (-122.58‒40.80)	21.41 (-57.72‒100.55)

a P<0.05.

Overall, there were few differences between intervention groups. There were no statistically significant differences in healthcare costs, sickness benefit costs, or outcome in SA days between any intervention group and TAU (table 2). Bootstrapping-created distributions of the ΔC and ΔE for ACT, WDI and ACT-WDI compared with TAU for one- and two-years follow-ups demonstrated major uncertainty of cost-effectiveness results (see supplementary figures S1–6).

**Figure 2:**
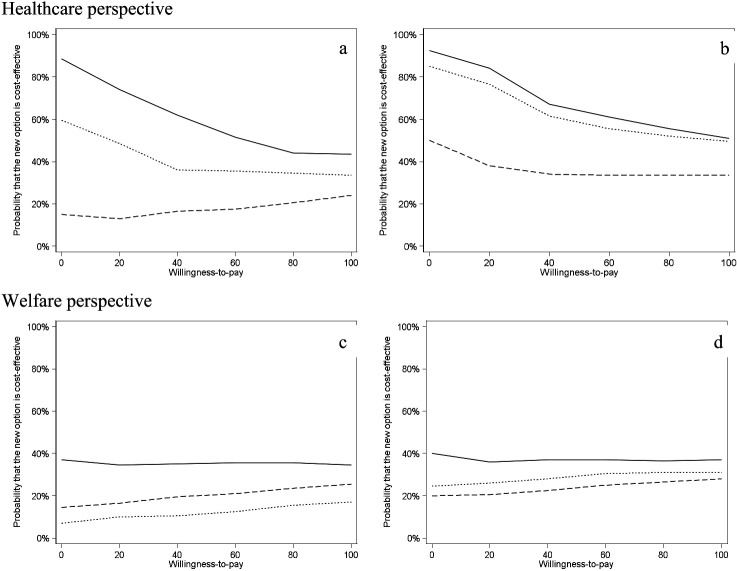
Cost-effectiveness acceptability curves for ACT, WDI, ACD+WDI compared with TAU over one- (left) and two (right) years follow-up. ACT – solid line, WDI - dashed line, ACT+WDI - dotted line.

Cost-effectiveness acceptability curves in figure 2 demonstrate the probability of the interventions to be cost-effective from the healthcare and limited welfare perspectives.

From a healthcare perspective (figure 2, a–b), there was a 90% probability of ACT being cost-saving compared with TAU over both the one- and two-year follow-up. For ACT+WDI, the probabilities of cost-saving compared to TAU were 60% for the first year, which increased to 85% for the two-year follow-up. The probabilities of ACT and ACT+WDI being cost-effective decreased with increasing WTP values. For WDI, the probability of being cost-saving was <50% for both one and two years. The low probability of WDI being cost-effective did not change with increasing WTP demonstrating broad uncertainty in the results.

From the limited welfare perspective (figure 2, c–d), none of the three treatment options exceeded 40% probability of cost-saving either for one- or two-year follow-up compared with TAU. Neither did the low probability of being cost-effective change with increasing WTP for any group.

### Subgroup analyses

When the analyses were conducted separately for two diagnostic subgroups – depression or anxiety disorders, and stress-induced exhaustion disorder – new patterns emerged.

**Figure 3:**
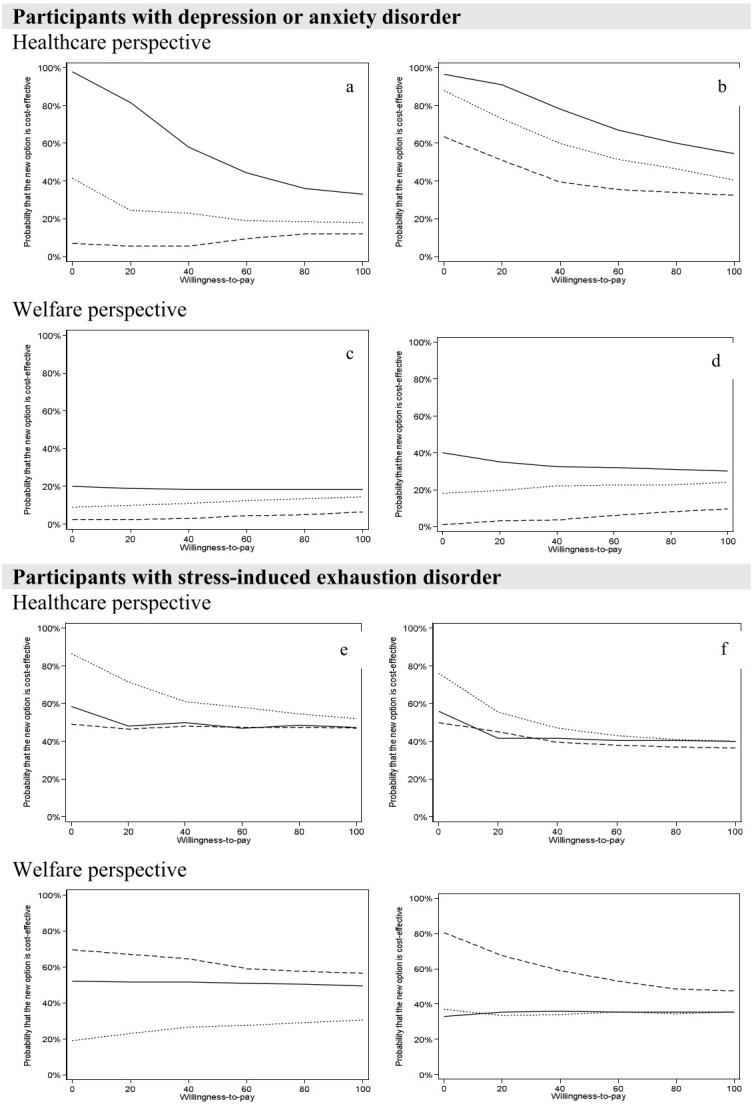
Cost-effectiveness acceptability curves for ACT, WDI, ACD+WDI compared with TAU over one- (left) and two (right) years follow-up for Depression and anxiety disorders and Stress-induced exhaustion disorder, respectively. ACT – solid line, WDI - dashed line, ACT+WDI - dotted line.

For the participants with depression or anxiety disorders, the ACT intervention significantly reduced healthcare costs during one- and two-years follow-up, but not SA days (see table 2). The WDI intervention generated significantly more SA days during the two-year follow-up. There were no significant differences in costs or SA days for ACT+WDA compared with TAU. From the healthcare perspective (figure 3, a–b), ACT had a very high probability (>99%) to be cost-saving for this sub-group of participants both at one-, and two-year follow-up. For ACT+WDI, the probability of being cost-saving increased from 40% in year one to 90% in year 2. Similarly, for WDI, there was an increase from 10% probability in year one of being cost-saving to 60% for the two-year follow-up. From the limited welfare perspective (figure 3, c–d) no intervention had higher than 40% probability of cost-saving compared with TAU.

For the participants with stress-induced exhaustion disorder (see table 2), WDI reduced SA days compared with TAU for both one- and two-year follow-ups (albeit not statistically significant). ACT+WDI significantly reduced healthcare costs during the first follow-up year but not during the two-year follow-up, compared with TAU. From a healthcare perspective (figure 3, e–f), there was an 85% probability of cost-saving in favor of ACT+WDI compared with TAU after one year. With a two-year time horizon, the probability of cost-saving decreased to about 75%. Bootstrapped results of the extra cost (ΔC) and extra effect (ΔE) for the participants in ACT+WDI with stress-induced exhaustion suggested high probabilities (about 80%) that the intervention could be both clinically superior and cost-saving during one- and two-year follow-up. The probability of ACT+WDI being cost-effective for this subgroup in year one remained around 60% as WTP increased. In contrast, ACT and WDI had lower probabilities of cost-saving for participants with stress-induced exhaustion disorder.

From the limited welfare perspective (figure 3, g–h), the WDI interventions had a 70% probability of being cost-saving in year one which increased to 80% over two years. ACT and ACT+WDI had a 50/20% probability of being cost-saving in year one/two, respectively, which reached 40% over two years for both groups compared to TAU.

An overall analysis, including both healthcare and welfare costs, is presented in the supplementary material.

## Discussion

This study reports on full economic evaluation of three RTW interventions for participants on SA with a diagnosis of depression, an anxiety disorder, or stress-induced exhaustion disorder, conducted alongside a randomized trial with a two-year follow-up. The results of the cost-effectiveness analyses from the healthcare and limited welfare perspectives for the total sample revealed no significant differences between the interventions. However, ACT demonstrated a high probability (90%) of reducing healthcare costs at both one-year and two-year follow-up. For ACT+WDI, the probability of healthcare costsaving improved from 60% in year one to 85% in year two. The probability of cost-effectiveness decreased with increasing WTP values, reflecting the broad uncertainty in the cost-effectiveness results. None of the interventions were more effective in reducing costs or SA, as compared with TAU. The subgroup analyses suggested important differences between diagnostic subgroups concerning healthcare costs. There was a high probability that the ACT intervention saves healthcare cost for patients with depression or anxiety disorders and ACT+WDI was the best alternative for stress-induced exhaustion disorder in terms of reduction in healthcare costs. From the welfare perspective, none of the treatment alternatives were better than TAU for depression or anxiety disorders. However, the WDI intervention was promising for stress-induced exhaustion disorder, mostly due to reduction in SA.

The results of this study supports the ICD-11 classification of stress-induced exhaustion disorder as an occupational phenomenon, given that a work-oriented intervention seems to better match the needs of participants with stress-induced exhaustion disorder and contributes to less SA. This is in line with a previous study that also found a short intervention focusing on ability to RTW cost-effective for a subgroup of participants with stress-related mental disorder (25). Altogether, this underscores the necessity to address work-factors for this type of mental health disorder.

For depression and anxiety disorders, ACT may reduce healthcare costs, potentially indicating that this therapy could be better suited to the needs of this subgroup. In line with previous research, psychotherapy seems to be a more cost-effective treatment option for depression from the healthcare perspective (26). However, there was no cost reduction in terms of sickness benefits. Based on the results from this study, the WDI should not be used for patients with depression and anxiety, given that it significantly increased SA days compared with TAU for this subgroup. A recently updated Cochrane review showed that stand-alone work-directed interventions may increase SA days for people with depression (11). The same review showed that psychological interventions might reduce SA days for depressed people, which was not the case in this trial; ACT did not reduce SA days compared to TAU for this subgroup of participants. In comparison, one study with a 27 month time horizon found psychotherapy in the workplace for depression to be the most cost-effective option (27) compared to pharmacotherapy and a combination of pharmacotherapy and psychotherapy.

Although SA due to common mental disorders implicates significant costs to societies, there are very few robust economic studies of RTW interventions (14), and follow-up is rarely longer than one year. The present study is one of the few economic evaluations of that kind of intervention with a longer time horizon and the first to examine the cost-effectiveness of ACT for mental disorders. The results of this study demonstrate the importance of long time horizons when evaluating the cost-effectiveness of RTW interventions given the differences in costs and effects between one- and two-year follow-ups.

In our previous economic evaluation alongside this randomized controlled trial of ACT, WDI, ACT+WDI, and TAU (19), we used one-year follow-up data and quality-adjusted life years (QALY) as health outcomes. We found that using QALY as the outcome measure, results are inconclusive (20). Moreover, using QALY may limit the usefulness of results if the end-user is an employer or non-health organization. An employer evaluating the effectiveness or cost-effectiveness of a new intervention would likely find SA days (the outcome used in the present study) to be a more relevant outcome.

In the present study, most of the differences between the intervention groups in both costs and effects were non-significant. In health economic evaluations, there is less emphasis on statistically significant differences between groups compared to other types of evaluations. Generally, most cost-effectiveness studies are underpowered. That is why we focused on the presentation of uncertainty based on estimation rather than hypothesis testing, using non-parametric bootstrapping, the incremental net-benefit statistic, and the presentation of cost-effectiveness acceptability curves (28).

### Limitations

SA days is a highly relevant outcome in insurance medicine and for employers, given that the primary cost driver is production loss when it comes to SA (29). In this study, we have considered costs from two perspectives; however, the employer perspective is not covered, which constitutes a limitation. Likewise, the study may be underpowered for the economic evaluation since power was calculated for the main outcome only (as is usual). CI for cost differences were very wide, which is a common challenge in economic evaluations conducted alongside RCT, caused by the small sample sizes based on detecting relevant differences in effects. Another limitation is the imbalance between diagnostic groups with a higher proportion of patients with stress-induced exhaustion disorder and fewer participants with depression and anxiety disorders. To address this, we stratified the analysis by the disorder.

The subgroup results suggest hypotheses for future studies. ACT stands out as a cost-effective treatment option in terms of healthcare costs for depression and anxiety disorders, but not for stress-induced exhaustion disorder. For stress-induced exhaustion disorder, WDI seems cost-effective from the welfare perspective, but not for depression or anxiety disorders. The cost-effectiveness of tailoring the treatment for individuals with different needs and diagnoses merit further research.

### Concluding remarks

This full economic evaluation shows that ACT may likely be cost-effective for employees on sick leave due to depression or anxiety disorders in terms of saving healthcare costs. Adding WDI to ACT seems to increase the cost-effectivenss of ACT for stress-induced exhaustion disorder. From the welfare perspective, stand-alone WDI interventions may be cost-effective for employees on sick leave due to stress-induced exhaustion disorder.

## Supplementary material

Supplementary material
