# Supplementary material for: Economic evaluation of return-to-work interventions for mental disorder-related sickness absence: two years follow-up of a randomized clinical trial
Source: Scand J Work Environ Health. 2022 Apr 29;48(4):264–72. doi: 10.5271/sjweh.4012 (PMC9524165; doi:10.5271/sjweh.4012)
Supplement: Supplementary material [file SJWEH-48-264-S001.pdf]

Economic evaluation of return-to-work interventions for mental disorder-related sickness absence: two years follow-up of a randomized clinical trial [1]

by Anna Finnes, PhD,[2] Jeffrey S Hoch, PhD, Pia Enebrink, PhD, JoAnne Dahl, PhD, Ata Ghaderi, PhD, Anna Nager, PhD, Inna Feldman, PhD

1. *Supplementary material*
2. *Correspondence to: Anna Finnes, Division of Psychology, Department of Clinical Neuroscience, Karolinska Institutet, Nobels väg 9, 171 77 Stockholm, Sweden. [E-mail: anna.finnes@ki.se]*

Supplement Content:

Table S1. Unit costs/visit, in Euro 2020

Table S2: Missing data (%) for the frequencies of health care visits

Supplementary Methods: Regression calculations for cost-effectiveness analyses

Figure S1-2. Bootstrapped results basecase analysis per intervention group 1 year and 2 years.

Figure S3-4. Bootstrapped results per intervention group for the subgroup analysis depression or anxiety disorders 1 year and 2 years.

Figure S5-6. Bootstrapped results per intervention group for the subgroup analysis stress-induced exhaustion disorder 1 year and 2 years.

Table S3. Mean differences in costs overall between treatment arms.

Figure S7. Cost-effectiveness acceptability curves for ACT, WDI, ACD+WDI compared with TAU over one- and two years follow-up for overall cost.

Figure S8. Cost-effectiveness acceptability curves for ACT, WDI, ACD+WDI compared with TAU over one- and two years follow-up for patients with depression or anxiety and with stress-induced exhaustion disorder for overall cost.

Table S1. Unit costs/visit, in Euro 2020

Professional	Costs
Medical doctor	389
Psychologist	275
Social worker	190
Physical therapist	137
Nurse	282

Table S2: Missing data (%) for the frequencies of health care visits to different professionals at all follow-up measurements

	Frequencies of health care visits to:				
	**Medical doctor**	**Psychologist**	**Social worker**	**Physical therapist**	**Nurse**
Post-treatment	20%	25%	22%	26%	22%
6-month FU	15%	10%	8%	9%	9%
12 -month FU	11%	5%	2%	4%	2%
24-month FU	17%	17%	17%	17%	17%

Regression calculations for cost-effectiveness analyses

Regression calculations were adjusted for the following covariates as: age, sex, education level, birthplace, previous grade of SA absence and marital status. Denoting these covariates as X and the treatment options as ACT, WDI, and ACT&WDI, the regression analysis estimated the coefficients for equations [1] and [2]

$$\text{Cost} = \alpha_0 + \alpha_{ACT}\,\text{ACT} + \alpha_{WDI}\,\text{WDI} + \alpha_{ACT\&WDI}\,\text{ACT\&WDI} + \alpha_{\mathbf{X}}\,\mathbf{X} \qquad [1]$$

$$\text{NDSA} = \beta_0 + \beta_{ACT}\,\text{ACT} + \beta_{WDI}\,\text{WDI} + \beta_{ACT\&WDI}\,\text{ACT\&WDI} + \beta_{\mathbf{X}}\,\mathbf{X} \qquad [2]$$

where the coefficient estimates of α_t and β_t equal the extra cost (ΔC) and extra effect (ΔE) estimates comparing treatment t to TAU (for t = ACT, WDI, and ACT+WDI).

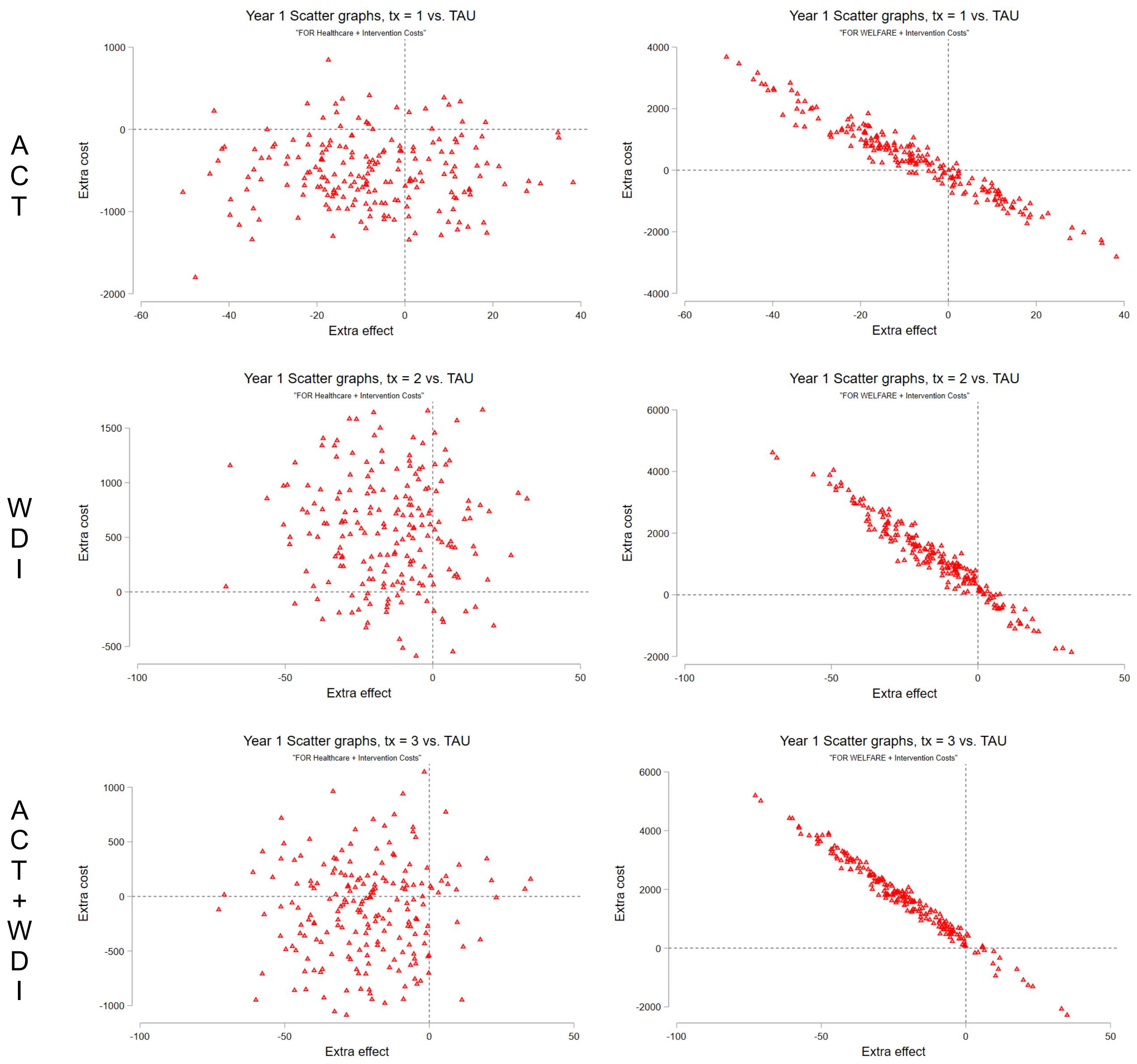

Figure S1. Cost-effectiveness planes for year 1 including all participants (n=264). The health perspective is presented to the right and the wellfare perspective to the left for each treatment arm compared to TAU.

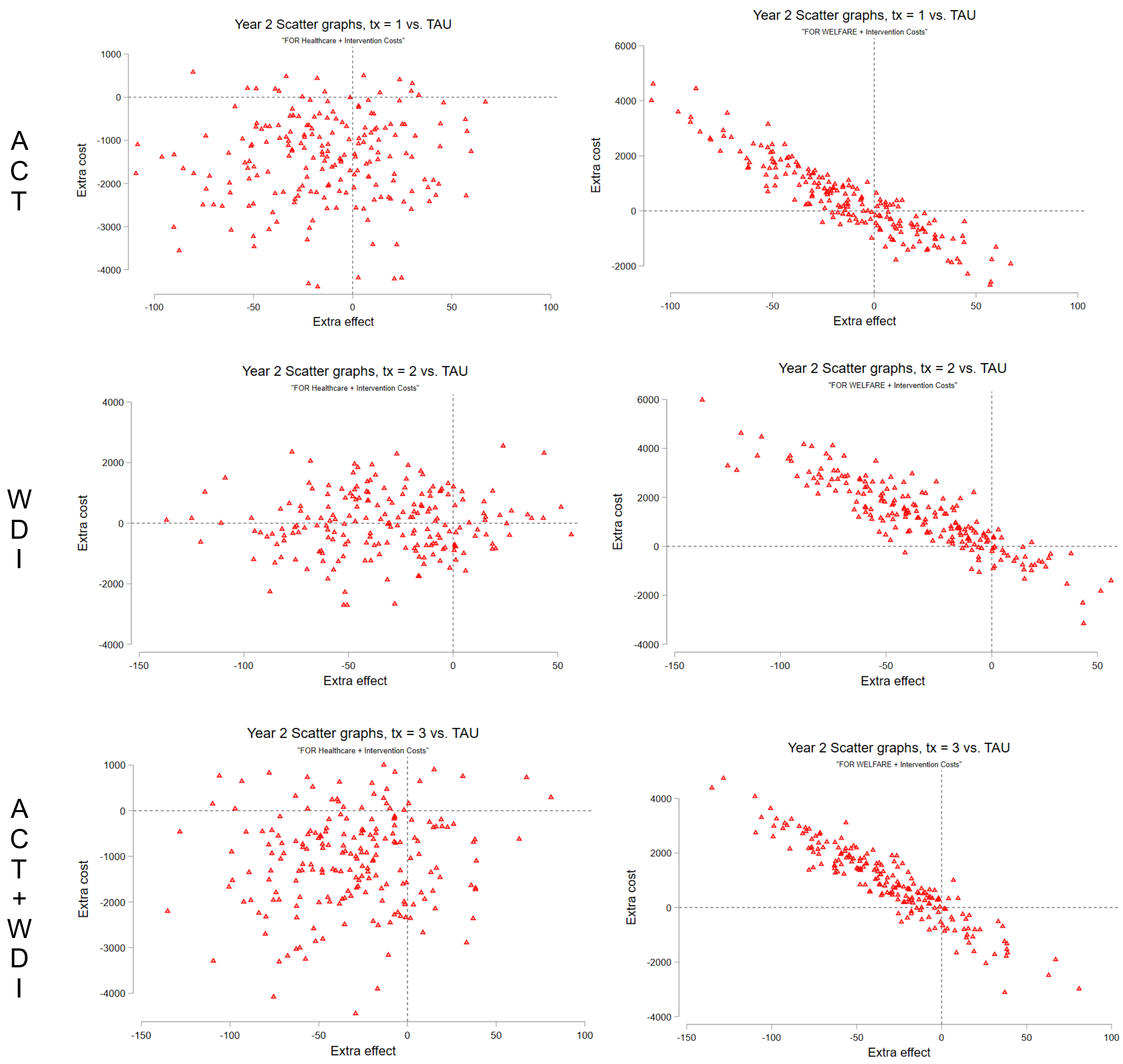

Figure S2. Cost-effectiveness planes for year 2 including all participants (n=264). The health perspective is presented to the right and the wellfare perspective to the left for each treatment arm compared to TAU.

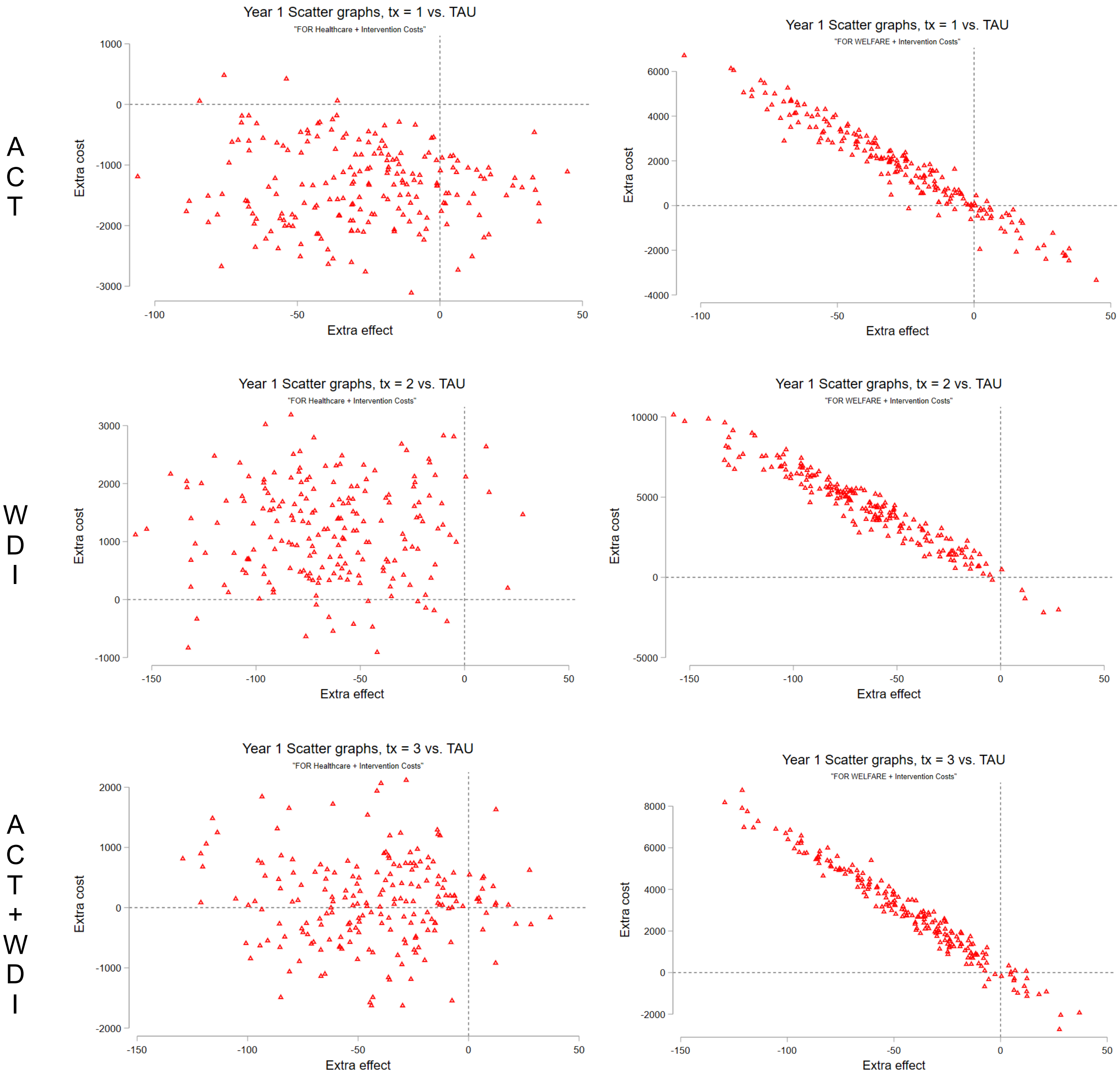

Figure S3. Cost-effectiveness planes for year 1 including participants with depression or anxiety disorders (n=107). The health perspective is presented to the right and the wellfare perspective to the left for each treatment arm compared to TAU.

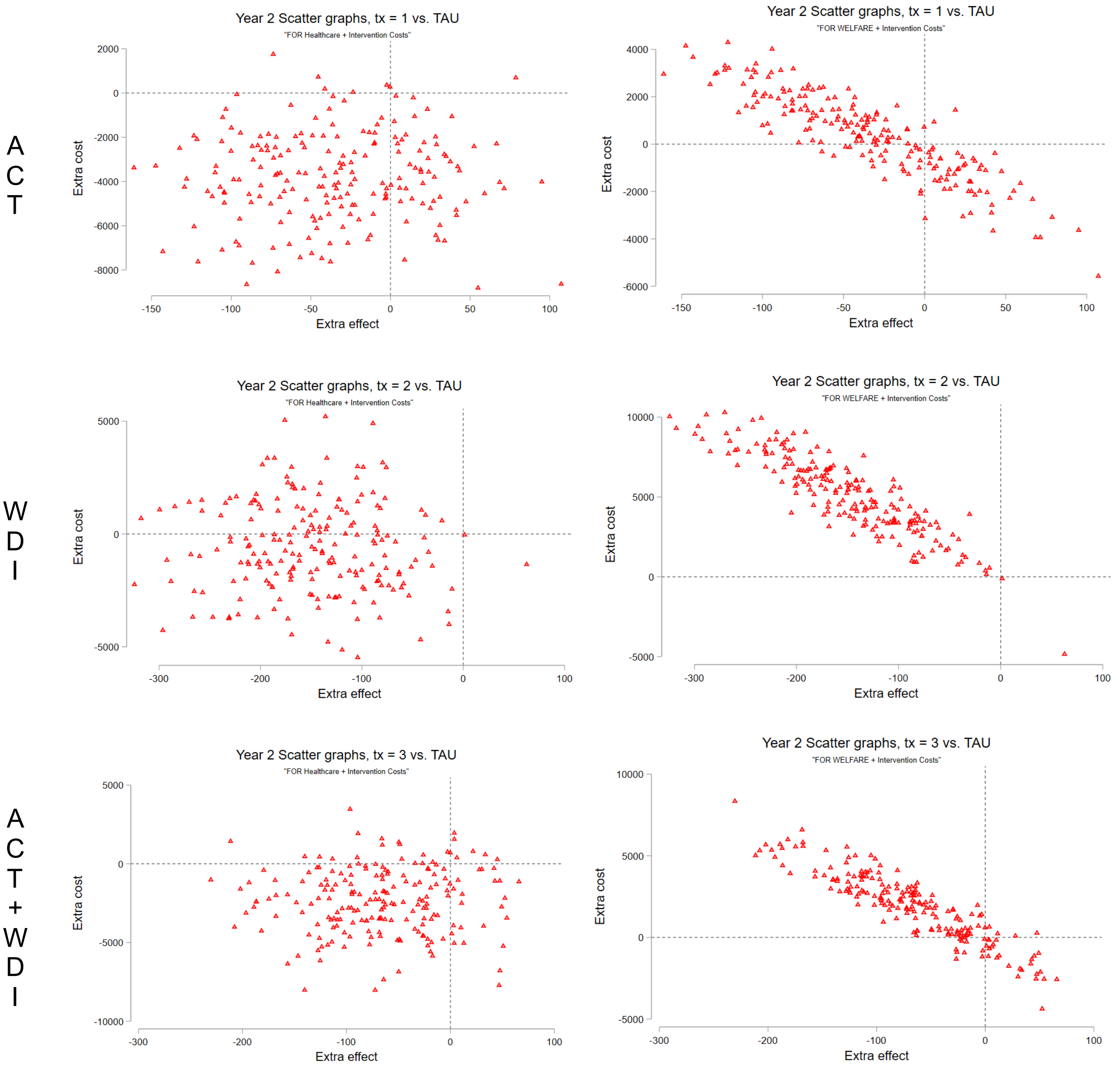

Figure S4. Cost-effectiveness planes for year 2 including participants with depression or anxiety disorders (n=107). The health perspective is presented to the right and the wellfare perspective to the left for each treatment arm compared to TAU.

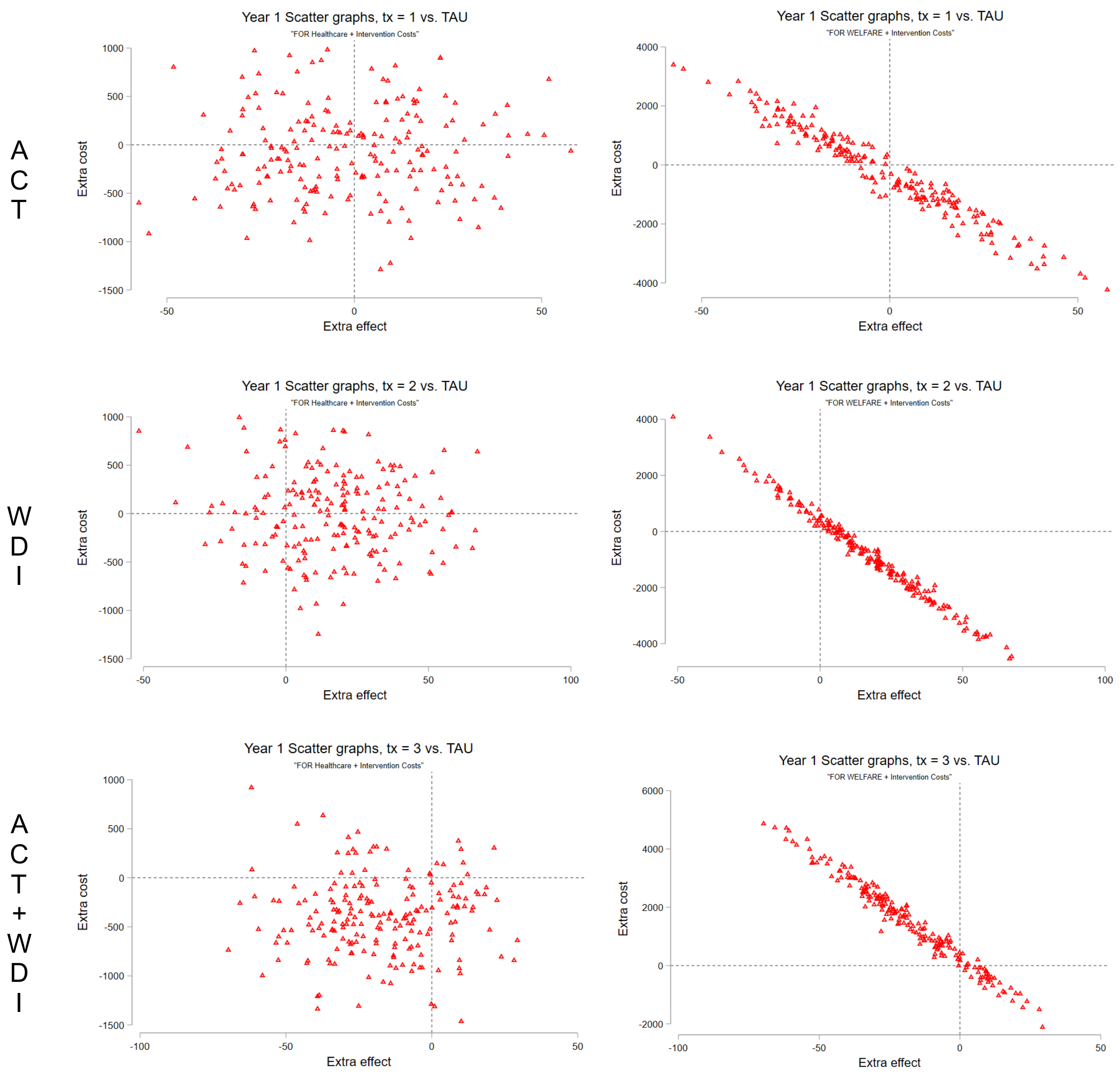

Figure S5. Cost-effectiveness planes for year 1 including participants with stress-induced exhaustion disorder (n=157). The health perspective is presented to the right and the wellfare perspective to the left for each treatment arm compared to TAU.

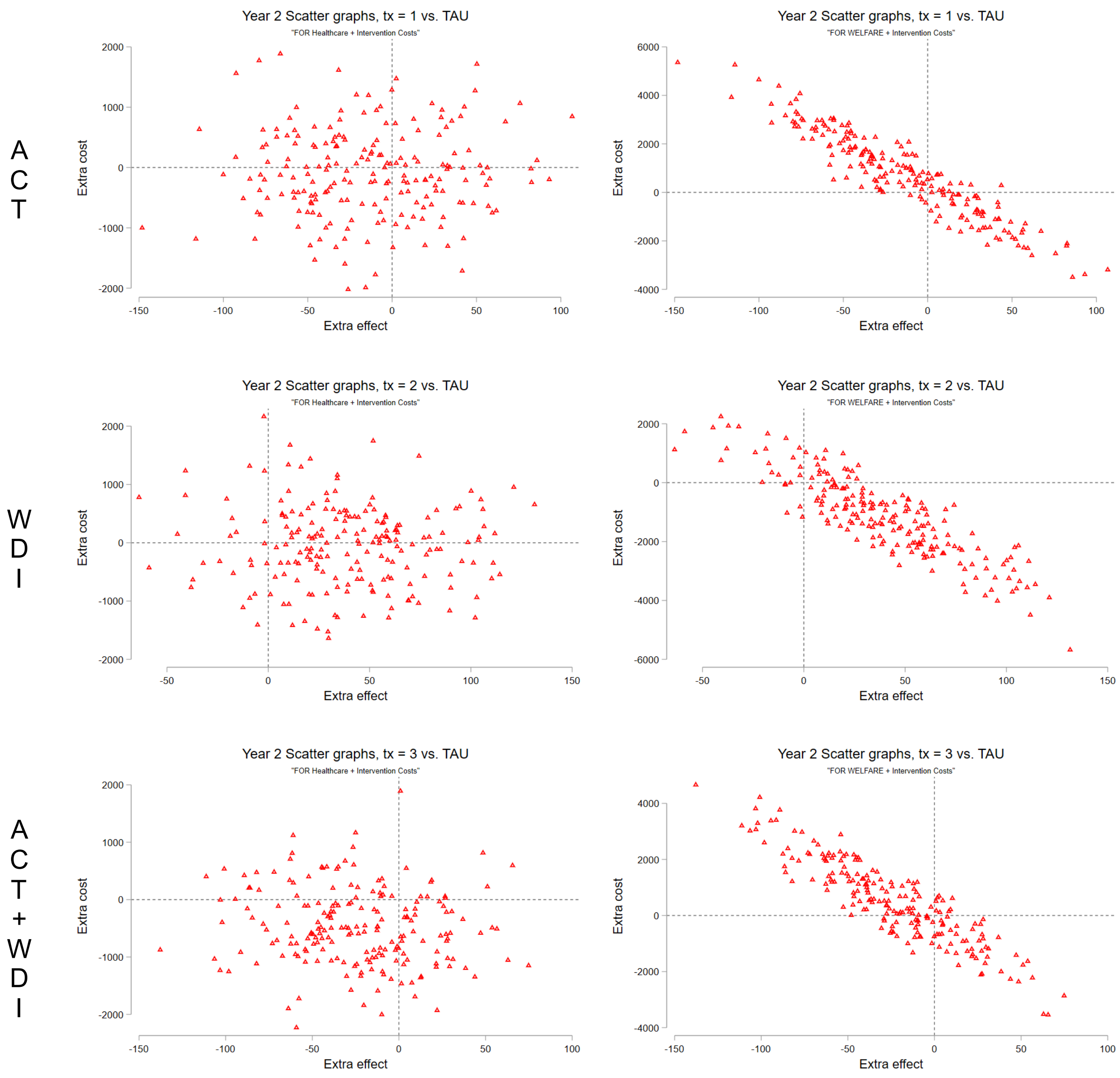

Figure S6. Cost-effectiveness planes for year 2 including participants with stress-induced exhaustion disorder (n=157). The health perspective is presented to the right and the wellfare perspective to the left for each treatment arm compared to TAU.

Table S3. *Mean differences in costs overall (in €2020) between treatment arms.*

Time horizon	ACT vs TAU<br>Estimate (95% CI)	WDI vs TAU<br>Estimate (95% CI)	ACT+WDI vs TAU<br>Estimate (95% CI)
	**All participants, n=264**		
Total cost 1 year	-423.04 (-2797.40–2081.58)	1275.95 (-1370.74–4202.75)	1112.67 (-1770.08–3463.33)
Total cost 2 year	44.34 (-4581.59–1773.43)	1706.776 (-2178.34–4137.29)	1450.44 (-4053.96–2644.72)
	**Participants with depression or anxiety disorder, n=107**		
Total cost 1 year	-372.58 (-3989.36–4080.86)	4439.41[a] (166.81–9789.97)	1961.72 (-2485.70–7796.48)
Total cost 2 year	-624.61 (-8548.14–1330.09)	6823.10 (-3286.88–9641.76)	1,676.33 (-7174.76–4369.68)
	**Participants with stress-induced exhaustion disorder, n= 157**		
Total cost 1 year	-730.17 (-3893.42–2069.29)	-1287.88 (-4387.09–1904.95)	282.72 (-2317.98–3478.00)
Total cost 2 year	10.19 (-3218.56–4061.16)	-2371.09 (-4692.58–1658.97)	1158.58 (-3850.64–2526.08)

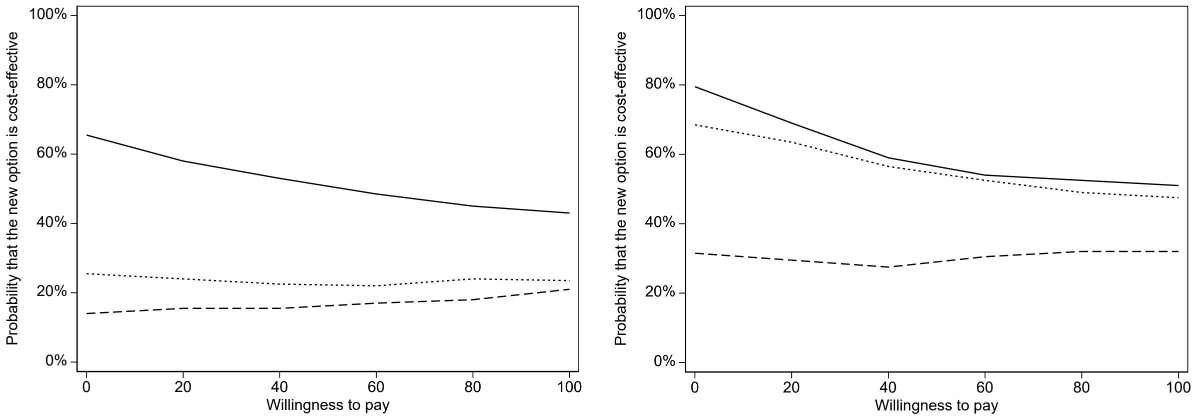

Figure S7. Cost-effectiveness acceptability curves for ACT, WDI, ACD+WDI compared with TAU over one- and two years follow-up. ACT – solid line, WDI - dashed line, ACT+WDI - dotted line.

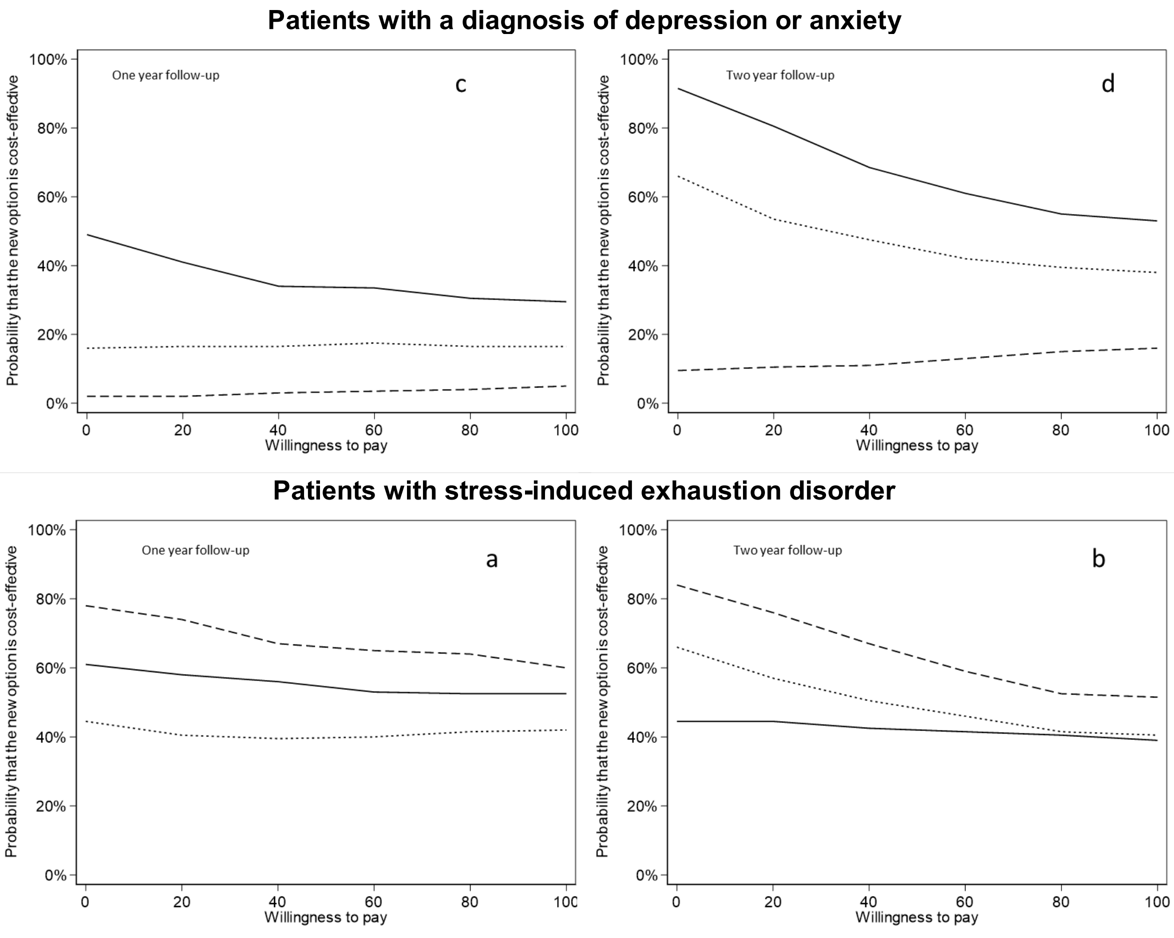

Figure S8. Cost-effectiveness acceptability curves for ACT, WDI, ACD+WDI compared with TAU over one- and two years follow-up for patients with depression or anxiety (c, d) and with stress-induced exhaustion disorder (a, b). ACT – solid line, WDI - dashed line, ACT+WDI - dotted line.
